# Do Early Childhood Educators Benefit from a Playful Working Environment? A Study on Workplace Fun and Work Engagement

**DOI:** 10.3390/bs15020149

**Published:** 2025-01-30

**Authors:** Jialing Yang, Xinghua Wang, Huifang Hong

**Affiliations:** 1 Faculty of Education, Beijing Normal University, Beijing 100875, China; yangjl@mail.bnu.edu.cn; 2 Department Name, Quanzhou Institute of Educational Science, Quanzhou 362000, China; huifanghong@126.com

**Keywords:** early childhood education teachers, workplace fun, psychological capital, work engagement

## Abstract

This study investigated early childhood education (ECE) teachers’ workplace fun and its relationship with their psychological capital and work engagement, both of which are crucial elements in improving their work efficiency. The relationship was investigated using quantitative methodology. Hypotheses and conceptual models were tested by structural equation modeling (SEM) on the raw data from 196 full-time ECE teachers via questionnaire surveys that investigated ECE teachers’ experiences of four aspects of workplace fun (i.e., formal fun activities, supportive practices for fun, coworker socializing, and fun job responsibilities). It was revealed that fun job responsibilities positively predicted the work engagement of ECE teachers. Furthermore, supportive practices for fun at ECE centers can indirectly predict the work engagement of teachers via the mediating effect of psychological capital. Based on the findings of this study, suggestions are provided to enhance teachers’ work engagement, including implementing supportive practices for fun by organizational management and promoting enjoyment in teachers’ job responsibilities.

## 1. Introduction

Play and playful pedagogy has been attributed significance for young children’s development, learning, and well-being since Friedrich Froebel’s time in the mid-nineteenth century. Creating a playful and inviting learning environment is an important aspect of early childhood education (ECE) teachers’ expertise (Hurme et al., 2023). Does play matter to adults as well? Would a playful working environment also benefit ECE teachers in terms of their working performance and well-being? Answering these questions is the fundamental motivation of conducting this study.

Increasing attention has been paid to ECE teachers’ professional well-being and their work engagement. According to previous research, the primary determinants of ECE teachers’ well-being and work engagement include job stress, demanding workload, lacking resources, psychological distress and an unfriendly work environment (Hakanen et al., 2006; Fan & Li, 2018; Angelini et al., 2024). In particular, teachers’ increased professional well-being was found to be correlated with better working conditions, supportive supervisors, coworkers socializing, greater professional autonomy (Kupers et al., 2022), higher job satisfaction, lower stress (Hur et al., 2016), and greater job commitment (Schreyer & Krause, 2016). However, research on enhancing the work environment and fostering a positive organizational culture of kindergartens remains inadequate. This study focuses on an important aspect of organizational culture, workplace fun, and explores its relationship with teachers’ work engagement.

The term “workplace fun” is used to describe a work environment that intentionally promotes enjoyable and pleasurable activities or one that elicits personal feelings of enjoyment, including formal fun activities, supportive practices for fun, coworker socializing, and fun job responsibilities (Wen et al., 2019; Plester & Hutchison, 2016). Formal fun activities are collective social activities designed to support emotional identification and pleasurable feelings (Ford et al., 2003; Tews et al., 2014), such as Teachers’ Day celebrations and team-building activities. Supportive practices for fun refer to the extent to which supervisors allow employees to relax (Michel et al., 2019), such as a management systems that support rest and relaxation. Coworker socializing includes friendly social interactions among colleagues, such as making jokes and sharing aspects of private life (Tews et al., 2014). Fun job responsibilities refer to undertakings that match individual interests (Tews et al., 2012, 2015). Workplace fun is a crucial and innovative approach to organization management, since an enjoyable and playful work environment has become a key motivator and vital incentive in young people’s career choices (Tews et al., 2012).

Workplace fun has a complex relationship with work engagement. Some research shows that workplace fun has a positive effect on employees’ work engagement (Plester, 2009; Becker & Tews, 2016). For instance, fun activities, coworker socializing, and manager support for fun all boosted workers’ emotional engagement (Bakker et al., 2011). Additionally, fostering a friendly workplace culture also increased employee engagement (Bakker et al., 2011). These studies considered fun as a social resource that helped people cope with work stress and engage in their work (Becker & Tews, 2016; Fluegge, 2008). Nevertheless, other research has shown that the associations between fun and work engagement may vary by context. An ethnographic study discovered that managed fun could result in some employees’ disengagement from the organization (Plester & Hutchison, 2016). Therefore, the relationship between workplace fun and work engagement is not merely a simple positive correlation, and its mechanism remains to be validated.

Workplace fun can be considered as a beneficial job resource (Demerouti et al., 2001). According to the Job Demands–Resources (JD-R) model, having access to appropriate job resources may facilitate attaining work-related objectives, meeting job requirements, and reducing job stress (Bakker & Demerouti, 2007), thereby fostering work engagement (Demerouti et al., 2001; Bakker et al., 2007). Studies have associated job resources with personal resources, such as psychological capital (Pierce et al., 1989). According to Xanthopoulou et al. (2009), psychological capital mediated the connection between various workplace resources (such as social support, supervisor coaching, autonomy, and career advancement possibilities) and work engagement as well as burnout. A study in China found that preschool teachers with high psychological capital had higher work engagement levels (Gao et al., 2023). Workplace fun, as a resource, may act on work engagement via fostering the psychological capital of kindergarten teachers.

ECE teachers’ emotional labor is distinguished by its extended duration, high intensity, and variety in emotional interactions (Zhang et al., 2020). Because of these characteristics, teachers often report feeling pressured and emotionally drained (Zhang et al., 2020). Workplace fun is a valuable resource that may help reduce the stress and boost energy. However, past research has focused on subjects such as remuneration, job satisfaction (Boyd, 2013; Abu Taleb, 2013; Schreyer & Krause, 2016), and work-related stress (Faulkner et al., 2016; Nislin et al., 2016), but research on ECE teachers’ working environment and its correlation with work engagement is lacking.

This study examines the relationship between workplace fun and work engagement from an organizational management perspective. Research questions comprised: (1) Can workplace fun of ECE teachers (formal fun activities, coworker socializing, supportive practices for fun, and fun job responsibilities) affect their work engagement? (2) Can workplace fun of ECE teachers affect their psychological capital, which further impacts on work engagement?

**Figure 1:**
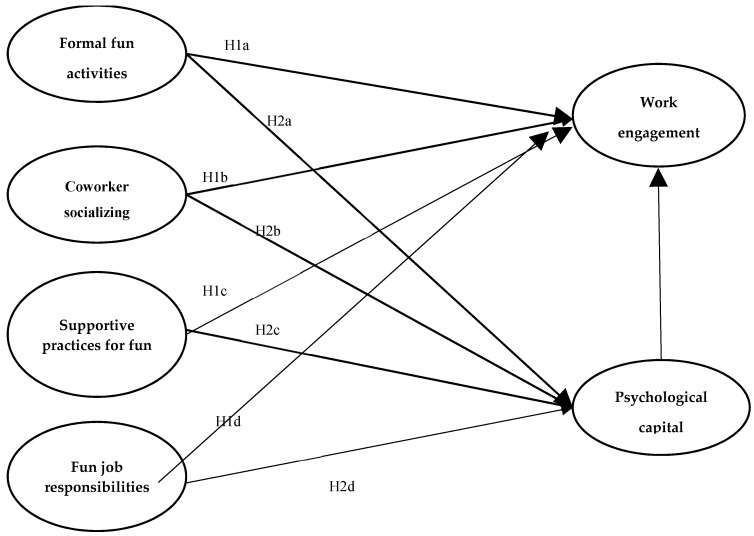
Hypothetical model of relationships among elements of workplace fun, psychological capital, and work engagement.

The hypothetical mode (Figure 1) is based on pertinent ideas and prior research on workplace fun. The model hypotheses were as follows:

**H1.**  *Workplace fun (i.e., (a) formal fun activities, (b) coworker socializing, (c) supportive practices for fun, and (d) fun job responsibilities) has a positive effect on ECE teachers’ work engagement.*

**H2.**  *If there is a relationship between workplace fun and work engagement, psychological capital of ECE teachers has a mediating effect on the relationship between workplace fun (i.e., (a) formal fun activities, (b) coworker socializing, (c) supportive practices for fun, and (d) fun job responsibilities) and teachers’ work engagement.*

## 2. Materials and Methods

### 2.1. Participants and Procedure

This research used convenience sampling. Survey questionnaires were delivered to 11 ECE centers across 9 districts in two cities in China’s Fujian Province. A total of 196 full-time ECE teachers completed the questionnaire voluntarily (Table 1). Teachers in the sample were aged 17 to 56, and 98.00% were female. Their teaching experience varied from two months to thirty-one years, and their monthly salaries ranged from CNY 1800 to CNY 8000 (about USD 252.9 to USD 1124). For educational background, 5 (2.6%) had a junior high school diploma or below, 41 (21.0%) a high school or secondary vocational school diploma, 39 (19.9%) a junior college degree, and 111 (56.7%) a bachelor’s degree or above. The following table also lists the quality ratings of the ECE centers, which were assessed by local educational quality regulation departments, including at provincial model level, municipal model level, district model level, and non-model level. The provincial model level indicated the highest quality, with the best performance in aspects including the environment quality, the teacher–child ratio, children’s development, and parent satisfaction.

### 2.2. Instruments

Workplace Fun. The Workplace Fun Scale was adapted by McDowell (2004), Tews et al. (2014, 2015), and Ford et al. (2003). Studies in China have already adapted these scales to Chinese versions (Yang et al., 2019; Shi & Yao, 2019). The 20-item Workplace Fun Scale used in this research had four dimensions: formal fun activities (FFA, 6 items), coworker socializing (CS, 4 items), supporting practices for fun (SPFF, 7 items), and fun job responsibilities (FJR, 3 items).

In this study, fun activities and coworker socializing were objective indicators, measured by frequency of occurrence over the previous year (Ford et al., 2003; Tews et al., 2015; Fu, 2015); supportive practices for fun and fun job responsibilities were based on teachers’ perceived experience, measured by the degree of perception over the previous year (Tews et al., 2014; Tews et al., 2015). As a result of interviews with ten ECE teachers, two items were added to the section on supportive practices for fun: “The supervisor understands and permits teachers’ asking for leave during work” and “The supervisor allows teachers to be flexible with their work schedule”. The wording of a few items was modified in line with the pre-surveys and interviews with ECE teachers. On a 5-point Likert scale, where 1 represents “never” and 5 represents “always”, higher ratings imply that kindergarten teachers have more workplace fun.

Work Engagement (WE). The scale of work engagement measured a positive, fulfilling work-related state of mind and it was modified from the Utrecht Work Engagement Scale-9 (UWES-9) developed by Schaufeli et al. (2006); a Chinese version has been utilized in prior studies (Lu & Luo, 2021). There were nine items overall, distributed throughout three sub-dimensions of vigor (3 items, e.g., “At my work, I feel bursting with energy”), dedication (3 items, e.g., “I am proud of the work that I do”), and absorption (3 items, e.g., “I am immersed in my work”) (Schaufeli et al., 2006). On a scale from 1 (never) to 7 (often), respondents were asked to rate their frequency of various features. Work engagement increased with the number of points.

Psychological Capital (PC). Based on research by Luthans et al. (2007), the 12-item Psychological Capital Questionnaire (PCQ-12) measured several aspects of psychological capital, such as hope (2 items), optimism (4 items), resilience (3 items), and self-efficacy (3 items) (Luthans et al., 2015, p. 242; Avey et al., 2011). The Chinese version’s wording (Luthans et al., 2015, pp. 221–222) was slightly modified to fit Chinese kindergarten teachers’ working conditions. On a six-point Likert scale, a score of 1 indicated “strongly disagree”, while 6 indicated “strongly agree”. Teachers with more psychological capital scored higher.

Control variables. Considering that work–family conflict and job satisfaction influence an individual’s work involvement (Wey Smola & Sutton, 2002; Saks, 2006; Zang & Feng, 2023), these two factors were accounted for as control variables. ECE teachers’ work–family time conflicts reflected time-based work interference with family. It was measured using a three-item Work-Family Conflict Scale modified from Carlson et al. (2000), with higher scores indicating a larger amount of time conflict between teachers’ work and family. One sample item was “My work keeps me from my family activities more than I would like”. The Job Satisfaction Scale measures an individual’s level of satisfaction with their job. It was derived from work by Ackfeldt and Wong (2006). It contains three items, and higher scores indicate greater job satisfaction. In addition, demographic factors such as age, education level, years of teaching experience, salary, and ECE centers’ quality levels were also investigated.

### 2.3. Data Analyses

To test hypotheses and examine the conceptual model, we performed structural equation modeling (SEM) using Smart-PLS 3.3.2 (SmartPLS GmbH, Bönningstedt, Germany). In contrast to covariance-based SEM (CB-SEM), the PLS-SEM evaluates models using variance-based partial least squares (PLS). For medium population effect sizes with a sample size of 100, and for weak population effect sizes with about 250 observations, PLS can attain a statistical power of 0.80 (Reinartz et al., 2009). There were two phases to the data analysis. The measurement model was first evaluated to check the reliability and validity of the research instruments. Second, the structural model tested the conceptual model’s pathways. To evaluate the structural model, we computed path coefficients and path loading significance. We used a bootstrap resampling method with 196 examples and 5000 sub-samples (Anderson & Gerbing, 1988; Wan & Shen, 2015).

## 3. Results

### 3.1. Assessment of the Measurement Model

Table 2 demonstrates that all factor loadings are greater than 0.5 and statistically significant at *p* < 0.001. The measurement model’s reliability and convergence validity were assessed using Cronbach’s α, composite reliability (CR), and average variance extracted (AVE). All constructs (latent variables) have Cronbach’s α above 0.80 and CRs above 0.80, indicating good reliability (Fornell & Larcker, 1981). Additionally, all AVEs are greater than 0.50, supporting the measurements’ convergent validity (Barclay et al., 1995). In terms of discriminant validity, the test results reveal that the reflective items in the model have cross-loadings that are lower than those of the corresponding factor loadings. This meets discriminant validity criteria (Chin, 1998, p. 321). According to Fornell and Larcker’s (1981) criteria, each dimension’s AVE is higher than the square of the correlation coefficient. Thus, all current research measures have sufficient discriminant validity.

### 3.2. Descriptive Analyses

Table 3 shows the results of descriptive statistical and correlation analyses. Psychological capital and work engagement are strongly and positively correlated with the four elements of workplace fun. Additionally, psychological capital and work engagement are positively correlated.

### 3.3. Assessment of the Structural Model

Since there were six variables significantly correlated to psychological capital or work engagement, they were used as control variables in subsequent structural equation modeling, including teaching experience, education levels, job satisfaction, work–family time conflicts, salary, and age. In the assessment of the structural model, the cross-validated redundancy (Q2), the standardized root mean square residual (SRMR), and the goodness-of-fit index (GoF) reflect how well the structural model fits the data. All Q2 values in this model are higher than 0 (Table 4), indicating that the model has predictive relevance for all endogenous constructs (Hair et al., 2017, p. 237). The model has a good model fit because the SRMR is 0.06, which is lower than the 0.08 criterion (Henseler et al., 2014). The model fit reaches a large effect size because the GoF is 0.747 (Wetzels et al., 2009).

Table 4 shows that 65.6% of the variance in psychological capital and 79.1% of the variance in work engagement can be explained. The f^2^ effect sizes of predictors vary from 0.000 to 0.407. Among them, fun job responsibilities contribute the most to the R^2^ value of work engagement (f^2^ = 0.407), and supportive practices for fun have the greatest effect on psychological capital (f^2^ = 0.032).

**Figure 2:**
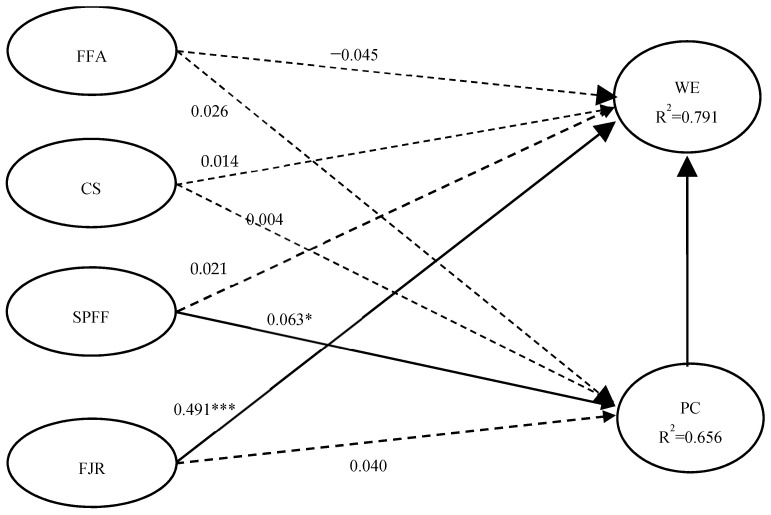
Standardized path coefficients of the structural model. Note: FFA = formal fun activities, CS = coworker socializing, SPFF = supporting practices for fun, FJR = fun job responsibilities, PC = psychological capital, WE = work engagement; dashed lines indicating non-significant path coefficients, solid lines indicating significant path coefficients; * *p* < 0.05, *** *p* < 0.001.

To evaluate the structural model, we estimated path coefficients and indirect effect coefficients, and then used a bootstrap resampling technique to examine the significance (Table 5). According to the PLS-SEM analysis, two of the eight hypotheses are supported by significant relationships at *p* = 0.001 and *p* = 0.05 levels (Table 5 and Figure 2). After controlling for the pertinent variables, fun job responsibilities were shown to have a positive and significant association with work engagement (*β* = 0.491, *p* < 0.001). The indirect effect’s path coefficient (SPFF→PC→WE) is significant (*β* = 0.063, *p* < 0.05), but the direct effect’s (SPFF→WE) is not (*β* = 0.021, *p* > 0.05). According to Hair et al. (2017, p. 233), this finding implies that psychological capital mediates the relationship between the supportive practices for fun and work engagement. Therefore, hypotheses H1d and H2c are supported, but H2d and H1c are not. In addition, formal fun activities and coworker socializing are not significantly associated with work engagement, either directly or indirectly, with no path coefficient significant at either *p* = 0.01 or *p* = 0.05 levels (see Table 5). Therefore, these results fail to support hypotheses H1a, H1b, H2a, and H2b. Figure 2 shows the structural model’s standardized path coefficients.

## 4. Discussion

### 4.1. The Positive Effect of Fun Job Responsibilities on Work Engagement

This study examined the effect of workplace fun on teachers’ work engagement across four dimensions (formal fun activities, coworker socializing, supportive practices for fun, and fun job responsibilities). It was discovered that fun job responsibilities predicted teachers’ work engagement, confirming previous research (Plester & Hutchison, 2016; Tsaur et al., 2019). Also, fun job responsibilities were more enticing than other purposely produced fun activities for job applicants (Plester et al., 2015). A very important aspect of ECE teachers’ work is to a create playful environment and interact with children in an enjoyable and supportive way. In turn, this is also a playful environment or fun job responsibility for teachers. Participating in playful daily activities is equally important for children as for teachers, which helps teachers develop positive attitudes about their jobs and become more engaged (Björklund & Elofsson, 2024; Bakker & Demerouti, 2007). This study also found that, when ECE teachers are confronted with the pressure of salary, social status, and a variety of other negative factors, the fun job responsibilities act as protective factors that help teachers engage in work positively.

### 4.2. The Indirect Effect of Supportive Practices for Fun on Work Engagement

This study suggests that supervisors’ fun-supportive practices boost employees’ psychological capital, which concurs with previous research. For instance, providing employees with a pleasant place to work increased their work enthusiasm, productivity (Deal & Kennedy, 1983), and satisfied their need for interests (Karl & Peluchette, 2006). In kindergartens, supportive practices for fun include promoting teachers’ fun at work, encouraging sufficient relaxation, and enabling schedule flexibility. These fun-supportive environments help teachers build harmonious relationships with supervisors and strengthen teachers’ feeling of control over their work speed, which boosts self-efficacy, initiative, hope, and resilience, all of which increase psychological capital.

According to the conservation of resources theory, an individual’s psychological capital, functioning as a personal resource, may affect their work status and behavior (Avey et al., 2009). Self-efficacy and optimism can help people handle stressful and high-demand situations (Hobfoll, 1989) and ultimately achieve positive results (Abbas et al., 2014; Karatepe & Karadas, 2014). Kindergarten teachers with higher psychological capital have more psychological resources to deal with stress or adapt their perspective while working under challenging conditions, helping them perform their job better and contribute to its success. Teachers who lack psychological capital may be more susceptible to stress caused by the depletion of psychological resources when faced with challenges. This can result in negative attitudes, such as dread of difficulties, disengagement, and laziness, which affects work engagement. Therefore, supportive practices for fun, as a positive job resource, can predict teachers’ work engagement by increasing psychological capital.

This study also shows that formal fun activities and coworker socializing have no significant influence on kindergarten teachers’ work engagement. Previous research on the effect of formal fun activity has been controversial. Although we referred to prior studies (Bakker et al., 2011; Becker & Tews, 2016; Fluegge, 2008; Tsaur et al., 2019) to make positive assumptions, existing qualitative studies (e.g., Redman & Mathews, 2002) have also found that there may be a negative relationship between formal fun activity and work performance. Redman and Mathews (2002) discovered that some people did not care about formal fun activities, and Fineman (2006) argued that an excessively rigorous administration of workplace fun may be problematic since spontaneity, surprise, and often subversion of the established order are what make fun “fun” (p. 280). An ethnographic study found that workplace fun can engage some people by providing breaks but distract others (Plester & Hutchison, 2016). Regarding this phenomenon, Plester et al. (2015) proposed the fun paradox, a dynamic equilibrium model that considers people in different work situations and positions and their complex fun needs.

Previous studies revealed that coworker socializing had a greater impact on recruiting appeal and retention than fun activities (Tews et al., 2012, 2014), but this study found no significant impact on kindergarten teachers’ work engagement. This is possibly because social friendships with coworkers may boost identity, organization embeddedness, and team integration (Kemph, 1969), but they may not directly affect teachers’ work engagement. Research shows that coworkers influence employees more when they engage in group activities (Chiaburu & Harrison, 2008). ECE teachers did have social connections with one another, but compared to service-oriented or social industries (e.g., restaurants) (Tews et al., 2014), their work is more autonomous and mostly independent, and it is therefore not evidently affected by coworker socializing.

### 4.3. Conclusions and Limitations

We explored the link between kindergarten teachers’ workplace fun and work engagement using the Job Demands–Resources model. This study found that kindergarten teachers’ work engagement was positively correlated with fun job responsibilities, and that its link with supportive practices for fun was mediated by psychological capital.

Since this research is a cross-sectional study, the association between workplace fun, psychological capital, and work engagement among kindergarten teachers can be determined, but their causal link is not established. Future research may include longitudinal or experimental studies of possible causal links between teachers’ workplace fun, psychological capital, and work engagement. In addition, this study surveyed ECE teachers’ experience of workplace fun throughout the previous year. However, because particular practices for fun (especially formal fun activities) are varied, the duration of their fun-producing effects may range for various activities. Future research could examine the immediate and long-term impacts of particular fun practices. Additionally, the relatively small sample size employed in this research may have restricted the generalizability of our findings. Future research could address this limitation by recruiting a larger and more diverse sample.

### 4.4. Implications

Results of this study confirm the value of creating playful and supportive working environments for ECE teachers. Playful pedagogy and playful curricula should be encouraged in preschool institutions, both from the perspective of early childhood development and from the perspective of teacher development. If teachers are encouraged to be agentic and work in an innovative way, they will find meaning and enjoyment in their work and make spontaneous adjustments to their job resources (Tims et al., 2012).

If we hope that teachers can have fun, and we should allow them to be themselves, because true fun is neither controlled nor constrained (Plester, 2009). To create a relaxing and harmonious psychological environment, preschool institutions could develop supportive practices for teachers’ fun, which have been shown to improve psychological capital and work engagement. First, given the fact that fun job responsibilities have positive effects on the work engagement of ECE teachers, the job content should match personal interests as much as possible. A career interest survey could also be used during the teacher recruitment process to screen candidates systematically. Moreover, ECE centers could allow expansion and flexible adjustment in individual teachers’ job responsibilities, such as arranging special projects, job rotations, and cross-training that fits teachers’ interests. Second, to develop supportive practices for teachers’ fun, ECE centers should encourage employees to have fun on the job, give teachers the freedom to take breaks or request time off when necessary, and allow autonomy in the design and implementation of playful pedagogy and playful curricula. Organizational culture building focusing on playfulness would allow teachers to have fun at work and contribute to their well-being (Georganta, 2012).

Some practices were not found to be significant in promoting ECE teachers’ psychological capital or work engagement. This also provides insight for kindergarten management. Firstly, colleague socializing in or outside of work or joking around with each other may not be significantly beneficial, since teachers’ jobs are often time-intensive. Secondly, formal fun activities initiated by the organization may impose pressure on employees inadvertently (Everett, 2011). To avoid this, ECE centers should take teachers’ needs into account when organizing formal fun activities and support voluntary participation or independent choices, thus ensuring that resource-expending formal fun activities can attain the maximum effect.

Many organizations struggle with improving work engagement. Due to severe psychological resource depletion, unmet needs can lead to mood issues and work burnout. This study proves that workplace fun benefits ECE teachers’ psychological health and work engagement. It has extended the present knowledge of ECE teachers’ working environment and revealed workplace fun’s positive implications.

## Figures and Tables

**Table 1:** Sample demographics (*N* = 196).

	Range/Frequency	Percent (%)	Mean	Standard Deviation
Age	17–56		31.11	8.72
Teaching experience (months)	2–372		9.47	9.06
Education levels:				
High school or below	46	23.47		
Junior college degree	39	19.90		
Bachelor’s degree or above	111	56.63		
Monthly salary (CNY)	1800–8000 (about USD 248.28–USD 1103.46)		3168.92 (about USD 455)	1148.20
ECE centers’ quality				
Provincial model level	66	33.67		
Municipal model level	35	17.86		
District model level	42	21.43		
Non-model level	53	27.04		

**Table 2:** Reliability and validity of latent variables and observed variables.

LV	Fornell–Larcker Criterion ^a^						OV	IL ^b^	α	CR	AVE
	FFA	CS	SPFF	FJR	PC	WE					
FFA	0.802						a1	0.790	0.891	0.915	0.643
							a2	0.861			
							a3	0.699			
							a4	0.826			
							a5	0.826			
							a6	0.799			
CS	0.584	0.840					b1	0.810	0.863	0.906	0.706
							b2	0.851			
							b3	0.888			
							b4	0.809			
SPFF	0.647	0.532	0.862				c1	0.873	0.942	0.953	0.743
							c2	0.899			
							c3	0.917			
							c4	0.920			
							c5	0.865			
							c6	0.718			
							c7	0.827			
FJR	0.438	0.390	0.673	0.915			d1	0.917	0.903	0.939	0.837
							d2	0.898			
							d3	0.930			
PC	0.461	0.404	0.646	0.654	0.819		hp1	0.838	0.955	0.961	0.671
							hp2	0.820			
							hp3	0.805			
							hp4	0.841			
							op1	0.805			
							op2	0.826			
							r1	0.703			
							r2	0.853			
							r3	0.764			
							s1	0.852			
							s2	0.851			
							s3	0.863			
WE	0.361	0.341	0.631	0.817	0.747	0.889	f1	0.906	0.966	0.971	0.790
							f2	0.770			
							f3	0.771			
							de1	0.951			
							de2	0.951			
							de3	0.903			
							v1	0.856			
							v2	0.930			
							v3	0.936			

Note: FFA = formal fun activities, CS = coworker socializing, SPFF = supporting practices for fun, FJR = fun job responsibilities, PC = psychological capital, WE = work engagement, LV = latent variables, IL = indicator’s loading, OV = observed variables, α = Cronbach’s alpha, CR = composite reliability, AVE = average variance extracted, Fornell–Larcker criterion for discriminant validity. ^a^ The value of the diagonal is the square root of AVE; ^b^ All the factor loadings were significant at *p* < 0.001.

**Table 3:** Descriptive statistics and correlation analysis of data.

Variables	Mean	S. D.	Min	Max	1	2	3	4	5	6	7	8	9	10
1 FFA	2.566	1.101	1	5										
2 SPFF	3.340	1.082	1	5	0.626 **									
3 CS	3.200	0.983	1	5	0.575 **	0.528 **								
4 FJR	3.684	0.990	1	5	0.419 **	0.674 **	0.377 **							
5 PC	3.561	0.822	1	5	0.428 **	0.635 **	0.380 **	0.646 **						
6 WE	5.033	1.418	1	7	0.345 **	0.628 **	0.332 **	0.814 **	0.742 **					
7 Teaching experience (months)	9.473	9.060	2	372	0.160 *	0.174 *	0.112	0.085	0.259 **	0.067				
8 Education level	14.747	1.843	9	16	0.149 *	0.024	0.138	−0.039	0.003	−0.190 **	0.338 **			
9 ECE centers’ quality	2.420	1.533	1	4	−0.234 **	−0.169 *	−0.085	−0.038	−0.042	0.068	−0.246 **	−0.549 **		
10 Job satisfaction	3.532	0.980	1	5	0.351 **	0.663 **	0.361 **	0.769 **	0.704 **	0.767 **	0.077	−0.127	0.013	
11 Work–family time conflicts	3.257	1.071	1	5	−0.169 *	−0.288 **	−0.104	−0.261 **	−0.193 **	−0.248 **	0.162 *	0.211 **	−0.238 **	−0.328 **

Note: *N* = 196; FFA = formal fun activities, CS = coworker socializing, SPFF = supporting practices for fun, FJR = fun job responsibilities, PC = psychological capital, WE = work engagement; * *p* < 0.05, ** *p* < 0.01.

**Table 4:** f^2^ effect sizes of predictors and the coefficient of determination (R^2^).

	Variables	PC	WE
f^2^	FFA	0.008	0.005
	CS	0.000	0.001
	SPFF	0.032	0.001
	FJR	0.014	0.407
	PC		0.191
R^2^		0.656	0.791
Q^2^		0.421	0.604

Note: FFA = formal fun activities, CS = coworker socializing, SPFF = supporting practices for fun, FJR = fun job responsibilities, PC = psychological capital, WE = work engagement.

**Table 5:** Path coefficients and their significances.

Hypothesis	Path	Coeff.	S.D.	t	95% CI		Inference
H1a	FFA→WE	−0.045	0.051	0.884	−0.149	0.050	Not supported
H2a	FFA→PC→WE	0.026	0.023	1.155	−0.011	0.078	Not supported
H1b	CS→WE	0.014	0.044	0.323	−0.068	0.101	Not supported
H2b	CS→PC→WE	0.004	0.021	0.204	−0.035	0.051	Not supported
H1c	SPFF→WE	0.021	0.058	0.356	−0.089	0.141	Not supported
H2c	SPFF→PC→WE	0.063	0.030	2.110 *	0.014	0.133	Supported
H1d	FJR→WE	0.491	0.058	8.454 ***	0.370	0.599	Supported
H2d	FJR→PC→WE	0.040	0.032	1.230	−0.022	0.107	Not supported

Note: FFA = formal fun activities, CS = coworker socializing, SPFF = supporting practices for fun, FJR = fun job responsibilities, PC = psychological capital, WE = work engagement; * *p* < 0.05, *** *p* < 0.001.

## Data Availability

The datasets presented in this article are not readily available because the data are part of an ongoing study. Requests to access the datasets should be directed to the corresponding author.
